# An 8-Week Web-Based Weight Loss Challenge With Celebrity Endorsement and Enhanced Social Support: Observational Study

**DOI:** 10.2196/jmir.2540

**Published:** 2013-07-04

**Authors:** Melinda J Hutchesson, Clare E Collins, Philip J Morgan, Robin Callister

**Affiliations:** ^1^School of Health Sciences, Faculty of HealthPriority Research Centre in Physical Activity and NutritionCallaghanAustralia; ^2^School of Education, Faculty of Education and ArtsPriority Research Centre in Physical Activity and NutritionUniversity of NewcastleCallaghanAustralia; ^3^School of Biomedical Sciences and Pharmacy, Faculty of HealthPriority Research Centre in Physical Activity and NutritionUniversity of NewcastleCallaghanAustralia

**Keywords:** weight loss, Internet, commercial sector, user engagement, retention

## Abstract

**Background:**

Initial engagement and weight loss within Web-based weight loss programs may predict long-term success. The integration of persuasive Web-based features may boost engagement and therefore weight loss.

**Objective:**

To determine whether an 8-week challenge within a commercial Web-based weight loss program influenced weight loss, website use, and attrition in the short term, when compared to the standard program.

**Methods:**

De-identified data for participants (mean age 36.7±10.3 years; 86% female) who enrolled in the Biggest Loser Club (BLC) (n=952) and the BLC’s Shannan Ponton Fast Track Challenge (SC) for 8 weeks (n=381) were compared. The BLC program used standard evidence-based website features, with individualized calorie and exercise targets to facilitate a weight loss of 0.5-1 kg per week (–500kcal/day less than estimated energy expenditure). SC used the same website features but in addition promoted greater initial weight loss using a 1200 kcal/day energy intake target and physical activity energy expenditure of 600 kcal/day. SC used persuasive features to facilitate greater user engagement, including offering additional opportunities for social support (eg, webinar meetings with a celebrity personal trainer and social networking) endorsed by a celebrity personal trainer. Self-reported weekly weight records were used to determine weight change after 8 weeks. A primary analysis was undertaken using a generalized linear mixed model (GLMM) with all available weight records for all participants included. Dropout (participants who cancelled their subscription) and nonusage (participants who stopped using the Web-based features) attrition rates at 8 weeks were calculated. The number of participants who accessed each website feature and the total number of days each feature was used were calculated. The difference between attrition rates and website use for the two programs were tested using chi-square and Wilcoxon Rank Sum tests, respectively.

**Results:**

Using GLMM, including weight data for all participants, there was significantly greater (*P=*.03) 8-week weight loss in SC (–5.1 kg [–5.5 to –4.6 kg] or –6.0%) compared to BLC participants (–4.5 kg [–4.8, –4.2] or –5.0%). Dropout rates were low and consistent across groups (BLC: 17 (1.8%) vs SC: 2 (0.5%), *P*=.08) and 48.7% (456/936) of BLC and 51.2% (184/379) of SC participants accessed the website at 8 weeks, with no difference between programs (*P*=.48). SC participants accessed the discussion forums, menu plans, exercise plans, and educational materials significantly more than BLC participants (*P*<.05).

**Conclusions:**

Using a short-term challenge with persuasive features, including online social support with endorsement by a celebrity personal trainer, as well as a greater energy balance deficit, within a commercial Web-based weight loss program may facilitate greater initial weight loss and engagement with some program components. The results support the need for a more rigorous and prospective evaluation of Web-based weight loss programs that incorporate additional strategies to enhance initial weight loss and engagement, such as a short-term challenge.

## Introduction

Recent systematic reviews suggest Web-based interventions facilitate modest weight loss [1-3], and participants’ engagement with program features are a key factor associated with success [1]. Krukowski et al have shown that individuals who were consistent users of a Web-based weight loss program during the initial program weeks were more likely to continue to use the program features and achieve significantly greater weight loss after 6 months [4]. Furthermore, greater weight loss at the beginning of treatment has been identified as a predictor of long-term weight loss success and weight loss maintenance [5]. Therefore, Web-based weight loss programs that engage participants and enhance weight loss during the initial stages of treatment may be more successful in the long term.

Web-based weight loss program providers are therefore exploring new ways to improve initial program success. This includes the use of persuasive technology [6], which may positively influence participant engagement [7]. However, a previous review of the use of persuasive features by six popular weight loss websites indicated that techniques to date may not be very persuasive [8] due to poor dialogue support, limited credibility support, and moderate primary task and social support. This suggests that greater focus on evaluating the effectiveness of persuasive features in Web-based weight loss interventions, and how they influence engagement and weight loss success, is required.

Therefore, the aim of this observational study was to determine whether an 8-week “challenge” version of a commercial Web-based weight loss program influenced weight loss, website use, and attrition in the short term, when compared to the standard commercial Web-based weight loss program. The 8-week challenge provided enhanced system credibility support through the use of a celebrity personal trainer to endorse the program and host additional opportunities for social support.

## Methods

### Participants and Study Design

Participants were adults aged 18-74 years with a body mass index (BMI) >18.5kg/m^2^ who subscribed to the standard Web-based weight loss program for at least the minimum subscription length of 12 weeks from June 27, 2011, to October 24, 2011, or the 8-week “challenge” version of the program, which began October 24, 2011. The subscription must have been the participants’ first for the commercial program, and those who did not pay for their subscription (eg, free promotional program trials) were excluded. The cost of a subscription in 2011 was AU$149 for SC. For BLC, the cost ranged from AU$19.95 per month if paid upfront for 12 months to AU$49.95 per month if paid monthly.

### Intervention

The commercial Web-based weight loss program was managed by SP Health Co, Australia. The standard program was The Biggest Loser Club (BLC) [9]. The short-term efficacy of the standard program, which is underpinned by social cognitive theory, incorporates key components of effective behavioral weight loss interventions, and includes persuasive features (Table 1), has been previously demonstrated [10]. The challenge version of the program was the Shannan Ponton Fast Track 8-Week Challenge (SC). Key program components are summarized in Table 1 and in Multimedia Appendices 1-4. SC included all features of BLC. However, to facilitate greater participant engagement SC drew on persuasive system design by offering enhanced system credibility support through the use of a celebrity personal trainer to endorse the program and host additional opportunities for social support. The celebrity personal trainer was Shannan Ponton, who is a qualified personal trainer on a national television program, “The Biggest Loser Australia”. The SC also used more “challenging” energy intake and expenditure targets (Table 1), with the goal of achieving greater initial weight loss.

**Table 1:** Description of the key components of the Biggest Loser Club (BLC) and Shannan Challenge (SC) programs linked to Persuasive Systems Design 4 categories.

Component	BLC	SC
**Diet and exercise recommendations**		
	Individualized calorie targets based on participants’ estimated total energy expenditure at enrollment based on their reported height, weight, and activity level and their desire to lose weight (–500kcal/day less than estimated expenditure) or maintain their weight [Primary Task Support].	Calorie target is 1200 calories for all participants. The exercise plan is 6 days/wk with aim to burn 600 calories/day [Primary Task Support].
**Self-monitoring**		
	Food and physical activity diaries to monitor calorie targets and search engines to facilitate entry of food data [Primary Task Support].	As per BLC
	Monitoring of reported body weight, waist and hip girths; graphical display of changes in data and body (BMI) silhouette. Participants were encouraged to “weigh in” once/wk [Primary Task & Dialogue Support].	As per BLC plus weight loss leader board with a prize for member who achieves the greatest percentage weight loss each week [Primary Task, Dialogue & Social Support].
**Feedback**		
	Daily and weekly calculations of energy balance and meeting recommended nutrient and food group targets from online diary [Dialogue Support].	As per BLC
	Automated (computer-generated) weekly personalized feedback on their dietary intake and exercise based on their diary entries, as well as their use or lack of use of the standard website features, and the level of success of their weight loss [Dialogue Support].	As per BLC
**Education materials**		
	Weekly menu plan and grocery list [Primary Task Support].	As per BLC
	Weekly physical activity plan [Primary Task Support].	As per BLC plus choice of a Home or Gym exercise program including video demonstrations from a celebrity personal trainer “Shannan” [System Credibility Support].
	Weekly tutorials, fact sheets, and challenges, which participants are prompted to access via a weekly email [Primary Task Support].	As per BLC
**Social support**		
	Discussion forum [Social Support]	Exclusive discussion forum where only Challenge members can post comments [Social Support].
	Historical online meetings hosted by an accredited practicing dietitian could be viewed by BLC participants [Social Support].	Weekly video blog with personal trainer “Shannan” [Social & System Credibility Support]. Weekly online meeting with personal trainer “Shannan” including a video and chat function where members post questions and personal trainer replies in writing in real-time [Social & System Credibility Support]. Facebook page where the personal trainer “Shannan” posts motivating messages, questions, or challenges [Social & System Credibility Support].
**Reminders/Prompts**		
	Participants received weekly reminders to weigh in via email or SMS [Dialogue Support].	As per BLC

### Data Collection and Measures

SP Health Co collected the data that were provided to the researchers in a de-identified form. Ethics approval for the study was obtained from the University of Newcastle Human Research Ethics Committee, NSW, Australia.

Participants’ pretreatment demographic (sex, age, and ethnicity) and anthropometric characteristics (weight and height) were captured from an enrollment survey. Participants’ self-reported height and weight were used to calculate BMI (weight in kilograms divided by height in meters squared), which was categorized as healthy (BMI 18.5-24.9 kg/m^2^), overweight (BMI 25-29.9 kg/m^2^), or obese (BMI ≥30 kg/m^2^).

Participants self-reported weights (in kilograms) recorded online weekly were used to determine weight change achieved after 8 weeks. Two types of attrition rates were calculated: dropout and nonusage [11]. Dropout attrition rates were calculated based on the number of participants who did not complete the program and therefore included participants who cancelled their subscription prior to completing 8 weeks. Nonusage attrition rates were calculated based on the number of participants who did not drop out but stopped using all Web-based features and did not resume use within the 8 weeks. To describe website use, we calculated the number of participants who accessed each website feature (overall access, food diary entries, exercise diary entries, forum views and posts, menu plan, exercise plan, weekly educational materials, and live webinars attended) and the total number of days each feature was used.

### Statistical Analysis

Data analysis was undertaken using Stata 11.0 (StataCorp). Basic descriptive statistics were used to describe the baseline characteristics and website usage data. Differences between the two programs were tested using chi-square test for categorical data, *t* tests if normally distributed continuous data, or Wilcoxon Rank Sum test if non-normal continuous data. To determine the weight change achieved from enrollment to 8 weeks, generalized linear mixed models (GLMM) were utilized containing available self-reported weight records for all participants. Baseline age and sex were controlled for in the analyses as potential confounders. A secondary sensitivity analysis was conducted to determine the robustness of the results from the GLMM approach, by imputing missing 8-week weight data using the last observation carried forward (LOCF) method. A linear regression model was fitted with weight at 8 weeks as the outcome variable, group as the predictor variable, and enrollment weight, age, and sex as covariates.

## Results

### Pretreatment Characteristics

Overall the study included 1334 individuals (953 BLC, 381 SC). Participants were predominantly female (86%), with an average age of 36.7±10.3 years, and half were of Anglo-Saxon descent (Table 2). At enrollment, SC participants had a lower mean BMI (30.6 vs 33.0 kg/m^2^, *P*<.001) than BLC participants.

### Attrition Rates

In total, 19 participants (1.4%) dropped out during the 8-week period with no significant difference in dropout rates between the two programs, but a possible trend of higher dropout rates among BLC participants: BLC 17 (1.8%) vs SC 2 (0.5%), *P*=.08. Nonusage attrition rates were 49.7% (n=653/1315) at 8 weeks. There was no significant difference in nonusage attrition rates between programs (*P*=.47) as 51.2% of BLC (480/936) and 48.8% of SC (185/379) participants stopped using all website features during the 8 weeks and did not return to use.

### Weight Loss

The primary analysis using GLMM resulted in a mean self-reported weight reduction of −4.6 kg (95% CI −4.9 to −4.4 kg) or −5.3% for all participants. SC participants self-reported significantly greater weight loss (–5.1 kg [–5.5 to –4.6 kg] or –6.0%) than BLC participants (–4.5kg [–4.8, –4.2] or –5.0%) after 8 weeks (*P*=.03) with small effect sizes (0.06 and 0.14 respectively).

The sensitivity analysis using LOCF gave a mean self-reported weight loss of −2.7 kg (−2.9 to −2.6 kg) or −3.0% after 8 weeks for all participants. SC participants self-reported significantly greater weight loss (–3.0 kg [–3.3 to –2.6kg] or –3.4%) than BLC participants (–2.6 kg [–2.9 to –2.4 kg] or –2.9%) after 8 weeks (*P*<.001), with small effect sizes (0.09 and 0.15 respectively). A significantly higher proportion of SC participants self-reported a weight loss of ≥5% after 8 weeks than the BLC participants (27.3% vs 22.6%, *P*=.02). See Table 3.

### Website Use

Website use for SC and BLC participants is described in Table 4. SC participants accessed and posted to the discussion form, viewed the program plan, menu plans, exercise plans, and educational materials more than BLC participants (Table 4). BLC participants made a significantly greater number of food entries and used the online diary on significantly more days than SC participants (Table 4). There were no significant differences in the number of weekly weigh-ins recorded or the number of exercise entries in the online diary between the two programs.

**Table 2:** Baseline characteristics of participants who enrolled in the BLC or the SC programs.

		Total (n=1334)	BLC (n=953)	SC (n=381)	*P* value^a^
**Gender, % (n)**					
	Female	85.9 (1146)	85.2 (812)	87.7 (334)	.22
Age in yrs, mean (SD)		36.7 (10.3)	36.5 (10.7)	37.1 (9.1)	.35
**Ethnicity, % (n)**					
	Anglo-Saxon	51.0 (680)	49.5 (472)	54.6 (208)	.55
	Other	18.8 (251)	19.9 (189)	16.3 (62)	
	Did not wish to respond	30.2 (403)	30.6 (292)	29.1 (111)	
					
**BMI (kg/m^2^), mean (SD)**		32.3 (7.0)	33.0 (7.1)	30.6 (6.5)	<.001
	Normal weight % (n)	12.4 (166)	10.3 (98)	17.9 (68)	<.001
	Overweight % (n)	31.8 (424)	29.1 (277)	38.6 (147)	
	Obese % (n)	55.8 (744)	60.7 (578)	43.6 (166)	

^**a**^Differences between programs tested using chi-square test for categorical data and *t* tests for continuous data.

**Table 3:** Weight change after 8 weeks for BLC and SC participants using GLMM and LOCF (all analyses controlled for baseline age and gender).

		Mean (95% CI)				Effect size, Cohen’s *d*	*P* value for difference between groups
		Total (n=1334)	BLC (n=953)	SC (n=381)	Difference between groups		
**Primary analysis, GLMM**							
	Absolute (kg)	-4.6 (-4.9, -4.4)	-4.5 (-4.8, -4.2)	-5.1 (-5.5, -4.6)	-0.6 (-1.2, -0.6)	0.06	.03
	Percentage (%)	-5.3 (-5.4, -5.1)	-5.0 (-5.2. -4.7)	-6.0 (-6.4, -5.7)	-1.1 (-1.5, -0.7)	0.14	<.001
**Sensitivity analysis, LOCF**							
	Absolute (kg)	-2.7 (-2.9, -2.6)	-2.6 (-2.9, -2.4)	-3.0 (-3.3, -2.6)	-0.3 (-0.5, -0.2)	0.09	.005
	Percentage (%)	-3.0 (-3.2, -2.8)	-2.9 (-3.1, -2.6)	-3.4 (-3.7, -3.0)	-0.5 (-0.7, -0.4)	0.15	<.001
**Percentage weight change category**							
	Weight gain, % (n)	3.9 (52)	4.7 (45)	1.8 (7)	N/A	N/A	.02
	0% to <5%, % (n)	72.1 (962)	72.6 (692)	70.9 (270)			
	5% to <10%, % (n)	20.8 (278)	20.0 (191)	22.8 (87)			
	10% or more % (n)	3.2 (42)	2.6 (25)	4.5 (17)			

**Table 4:** Website use by BLC and SC participants from enrollment to 8 weeks.

	BLC (n=953)		SC (n=381)		*P* value	
	Percentage used, % (n)	Frequency^a^ (median IQR)	Percentage used, % (n)	Frequency^a^ (median IQR)	Percentage used^b^ %	Frequency^c^
Weekly weigh-in	97.8 (932)	5 (2-8)	97.6 (372)	5 (2-8)	.86	.83
Online diary—food	86.3 (822)	11 (3-28)	80.6 (307)	8 (1-25)	.009	.006
Online diary—exercise	77.7 (740)	4 (1-11)	73.2 (279)	5 (0-14)	.09	.46
Discussion forum posts	39.9 (380)	0 (0-2)	73.8 (281)	5 (0-22)	<.001	<.001
Discussion forum views	19.6 (187)	0 (0-0)	55.4 (211)	1 (0-8)	<.001	<.001
Accessed menu plan	91.0 (867)	3 (1-9)	96.3 (367)	8 (3-15)	<.001	<.001
Accessed physical activity plan	84.2 (802)	2 (1-4)	94.8 (361)	6 (3-11)	<.001	<.001
Accessed weekly educational tips and challenges	88.9 (847)	2 (1-5)	93.4 (356)	5 (2-8)	.01	<.001
Attended weekly online meeting (webinar)	N/A	N/A	57.0 (217)	1 (0-2)	N/A	N/A

^a^Frequency is the number of days the feature was used.

^b^Difference between the two programs tested using chi-square test.

^c^Difference between the two programs tested using Wilcoxon Rank Sum test.

## Discussion

### Principal Findings

The primary aim of this study was to determine whether an 8-week challenge version of a commercial Web-based weight loss program, which integrated persuasive features including system credibility support through the use of a celebrity personal trainer to endorse the program and host additional opportunities for social support, demonstrated greater initial weight loss and program engagement compared to the standard program. In the current study, the 8-week challenge version facilitated greater weight loss and engagement, but dropout and nonusage attrition rates were comparable to the standard program.

The true weight loss achieved by participants is likely to be in the range between the GLMM and LOCF results, that is, -3.4% to -6.0% for the SC and -2.9% to -5.0% for BLC, due to reasons previously described [12]. At the group level, the difference in weight loss between the groups was statistically different, but the effect size was small, suggesting that the difference between the two groups weight loss may not be clinically significant. Although there was no significant difference in attrition rates between the two programs, there was a trend for lower dropout attrition for SC participants. Furthermore, frequency of use of the website was superior for SC participants, and they were more likely to use some website components. SC participants engaged more with the standard social support components (eg, discussion forum) SC participants were also more likely to access educational materials than BLC.

The differences in initial weight loss and engagement between the BLC and SC programs could be partly explained by the inclusion of additional persuasive features. By offering more opportunities for social support to SC participants, a more supportive environment may have been created [13]. The support environment may have allowed SC participants to compare their performance to others (social comparison) or motivated participants to change their behavior if they recognized that other participants were successfully making change (social facilitation). Furthermore, the use of a celebrity personal trainer to endorse and provide content within the SC program may have been more persuasive by boosting the perceived credibility of the program. Alternatively, the differences in weight loss and engagement may be due to the more stringent energy intake and expenditure targets set as part of the SC program. For example, greater access to the educational materials may have been required to facilitate adherence with the targets, suggesting that these resources may be necessary to facilitate adherence to the energy intake and expenditure targets set as part of SC program. However, as compliance to the recommended energy intake and expenditure targets for each program were not measured and an observational study design was used, we cannot be sure which components of SC lead to greater engagement and weight loss (ie, the persuasive features of social support and/or system credibility support, or different energy expenditure and intake goals).This could be examined in future studies.

### Limitations

The weight loss analysis used participants’ weigh-in records self-reported online. However, the accuracy of weight self-reported on the Internet has been shown to be reasonable [14]. Outcomes were evaluated only during the 8-week challenge; therefore, the long-term impact on user engagement and weight loss were not considered. As the SC program was more expensive for participants to subscribe to than the BLC program, future studies should also consider the cost-effectiveness of the programs when evaluating program effectiveness. Finally, the use of a celebrity personal trainer also limits the external validity of the results.

### Conclusion

This preliminary observational study supports the need for further evaluation of Web-based weight loss programs that incorporate persuasive strategies, including enhanced credibility support and social support, to enhance initial weight loss and engagement. Future randomized control trials accompanied by mediation analyses should specifically determine which intervention components (ie, persuasive features: social support and/or celebrity endorsement, or stringent eating and physical activity recommendations) of the Web-based program are associated with improvements in engagement and weight loss, and whether initial weight loss and engagement are maintained in the long term.

## Multimedia Appendix 1

Online food and exercise diary used by SC and BLC participants.

## Multimedia Appendix 2

Weekly self-monitoring of weight for BLC and SC.

## Multimedia Appendix 3

Additional opportunity for social support for SC participants: Facebook page.

## Multimedia Appendix 4

Additional opportunity for social support for SC participants: Weekly online meeting.

## Abbreviations

BLC: Biggest Loser Club
BMI: body mass index
GLLM: Generalized Linear Mixed Model
LOCF: last observation carried forward
SC: Shannan Ponton Fast Track Challenge
