# Supplementary figures and images for: An 8-Week Web-Based Weight Loss Challenge With Celebrity Endorsement and Enhanced Social Support: Observational Study
Source: J Med Internet Res. 2013 Jul 4;15(7):e129. doi: 10.2196/jmir.2540 (PMC3713892; doi:10.2196/jmir.2540)

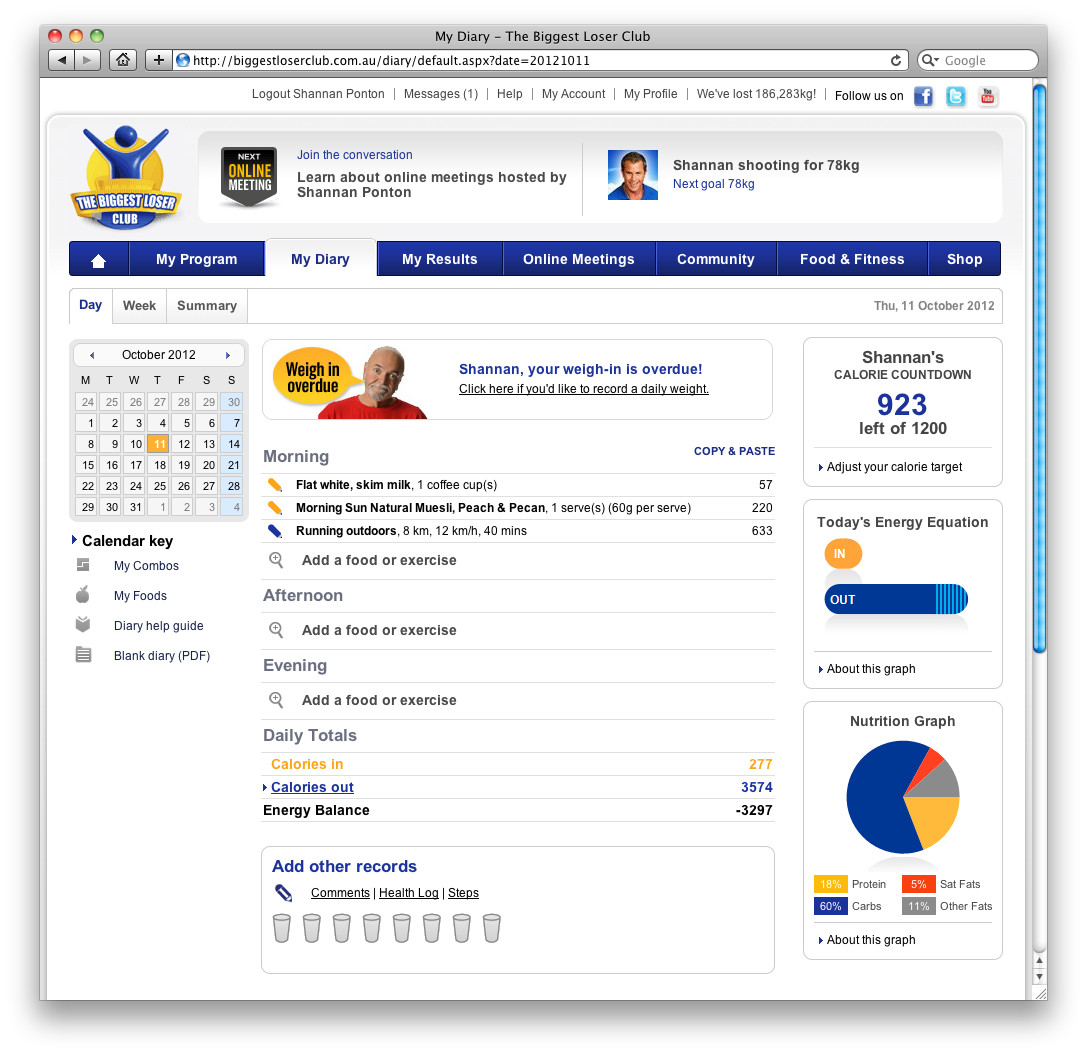

Supplement: Supplementary file 1 [file jmir_v15i7e129_app1.jpg]

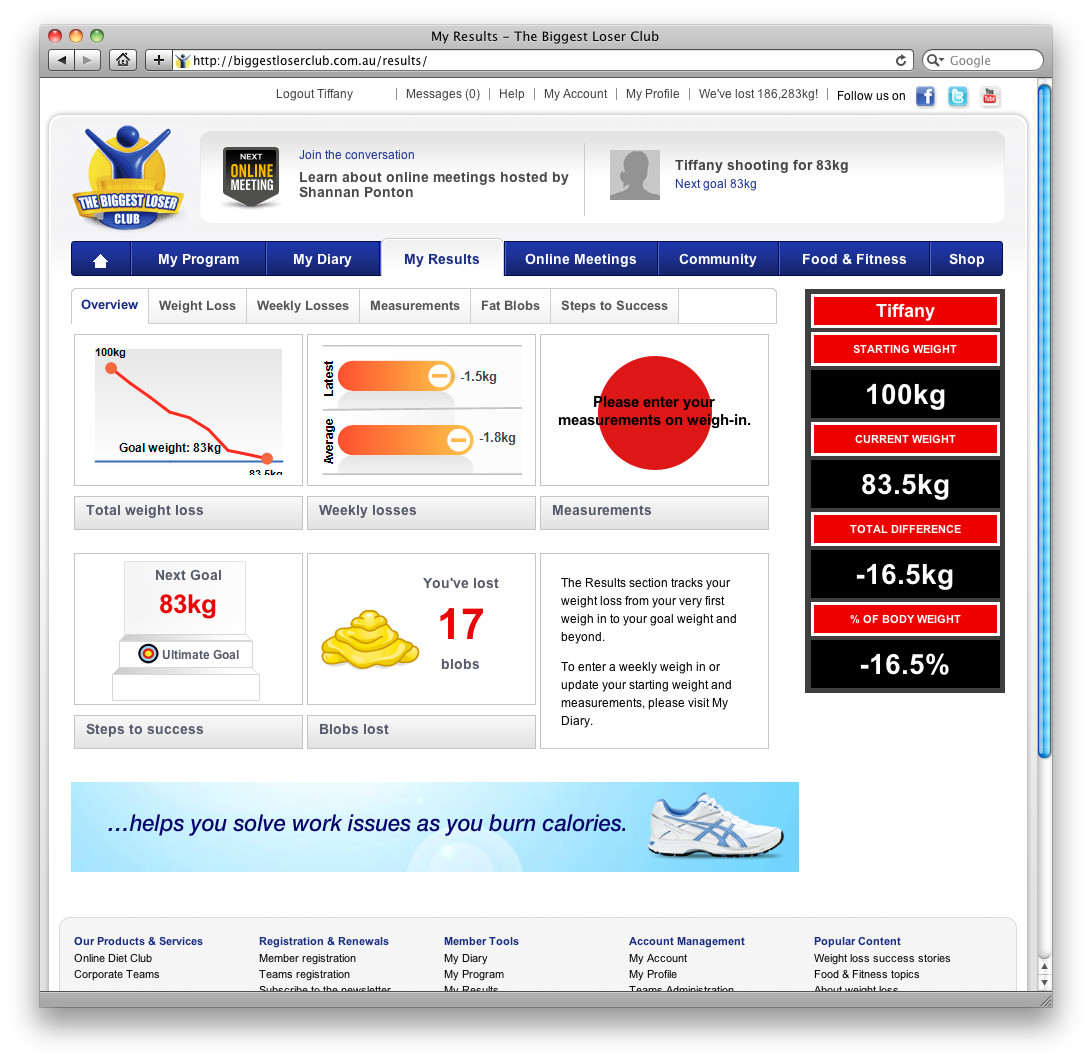

Supplement: Supplementary file 2 [file jmir_v15i7e129_app2.jpg]

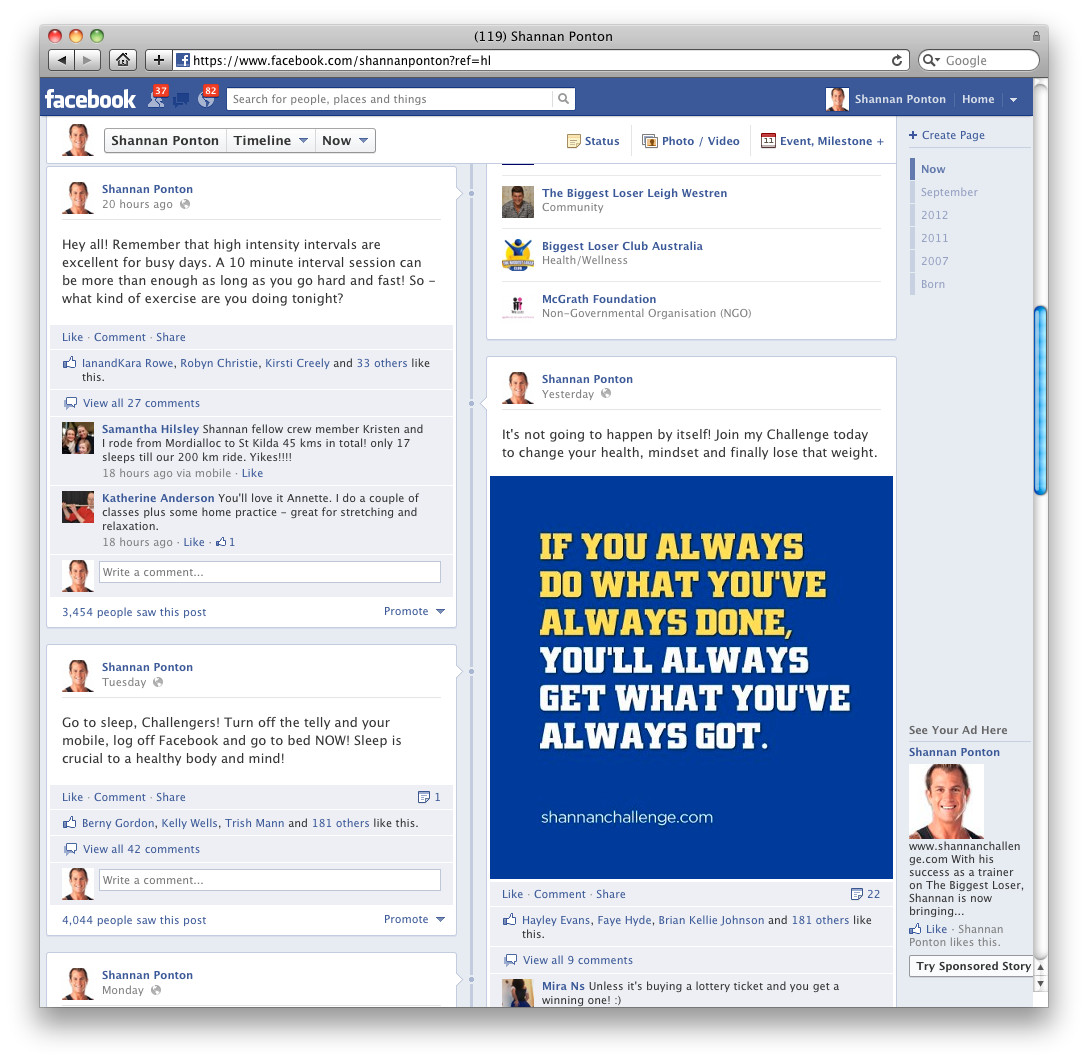

Supplement: Supplementary file 3 [file jmir_v15i7e129_app3.jpg]

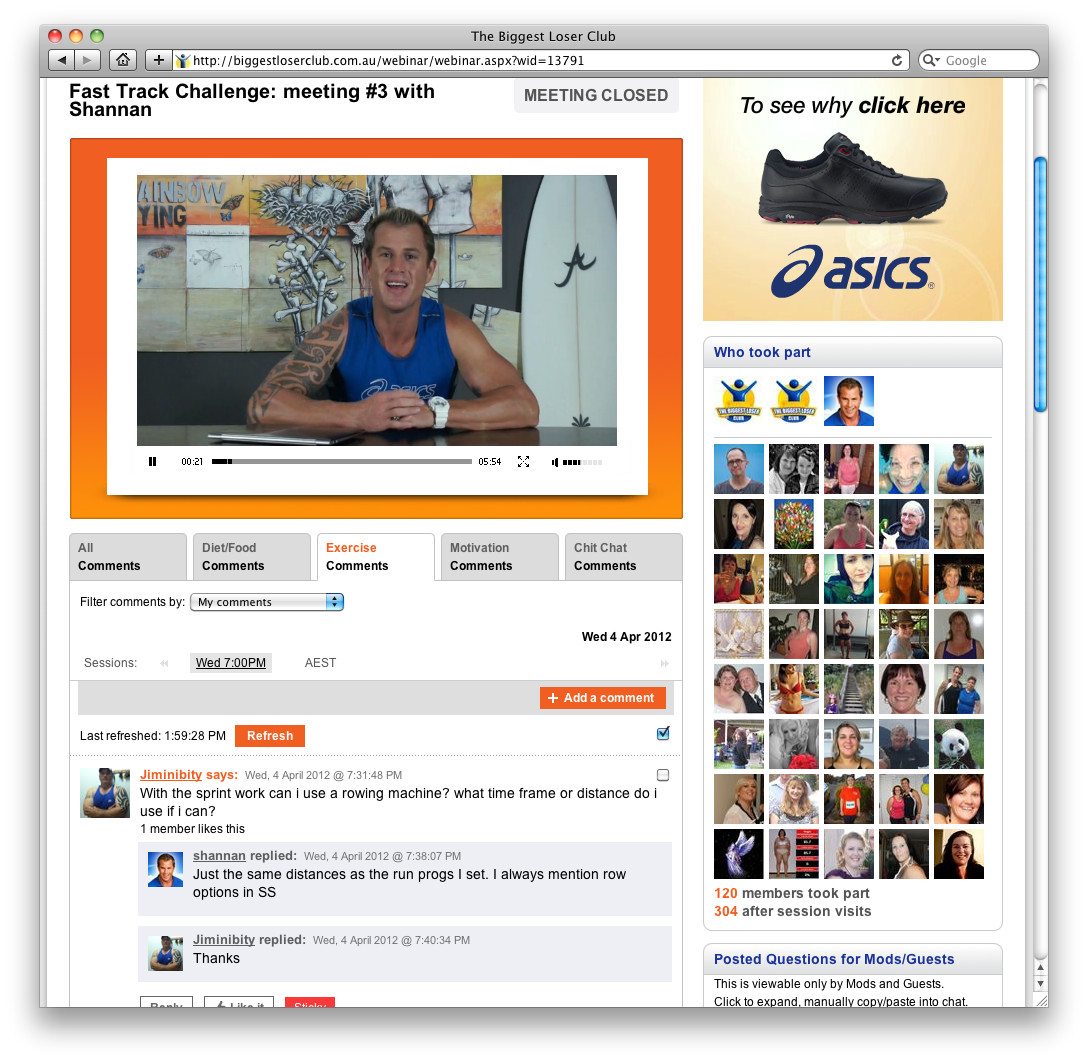

Supplement: Supplementary file 4 [file jmir_v15i7e129_app4.jpg]
